# Prevalence of Pulmonary Nematodes in Cats and Lung Ultrasound Findings in Separate Animal Cohorts: A Coprological, Molecular and Clinical Study

**DOI:** 10.3390/ani16040622

**Published:** 2026-02-15

**Authors:** Dawid Jańczak, Agata Moroz-Fik, Karolina Radziejewska, Aleksandra Kornelia Maj, Piotr Górecki, Jakub Kędziorek, Mateusz Antecki, Anna Maria Pyziel, Olga Szaluś-Jordanow

**Affiliations:** 1 Department of Infectious and Invasive Diseases and Veterinary Administration, Institute of Veterinary Medicine, Faculty of Biological and Veterinary Sciences, Nicolaus Copernicus University, Lwowska Str. 1, 87-100 Toruń, Poland; djanczak@umk.pl; 2 Division of Veterinary Epidemiology and Economics, Institute of Veterinary Medicine, Warsaw University of Life Sciences-SGGW, Nowoursynowska Str. 159c, 02-776 Warsaw, Poland; agata_moroz@sggw.edu.pl (A.M.-F.); karolina_radziejewska@sggw.edu.pl (K.R.); 3 Animallab Veterinary Laboratory, Środkowa Str. 2/4, 03-430 Warsaw, Poland; aleksandrakorneliamaj@gmail.com (A.K.M.); piotrg730@gmail.com (P.G.); jakubkedziorek2@gmail.com (J.K.); mateuszantecki2@gmail.com (M.A.); 4 Department of Public Health Protection and Animal Welfare, Institute of Veterinary Medicine, Faculty of Biological and Veterinary Sciences, Nicolaus Copernicus University, Lwowska Str. 1, 87-100 Toruń, Poland; anna.pyziel@umk.pl; 5 Department of Small Animal Diseases with Clinic, Institute of Veterinary Medicine, Warsaw University of Life Sciences-SGGW, Nowoursynowska Str. 159c, 02-776 Warsaw, Poland

**Keywords:** pulmonary nematodes, *Aelurostrongylus abstrusus*, *Eucoleus aerophilus*, *Troglostrongylus brevior*, Poland

## Abstract

Background: Pulmonary nematodes are an underrecognized cause of respiratory disease in domestic cats, with diagnosis often complicated by nonspecific clinical signs and limitations of fecal-based testing. Methods: This study aimed to assess the prevalence of feline lungworms in Poland and to describe lung ultrasound findings in a separate clinical cohort of cats. A nationwide coprological survey was conducted using pooled fecal samples from 1058 cats examined with Baermann and flotation techniques, supported by molecular diagnostics where available. Results: Overall, 9.83% of cats were positive for at least one parasite. *Aelurostrongylus abstrusus* was the most frequently detected lungworm (7.18%), followed by *Eucoleus aerophilus* (2.17%) and *Troglostrongylus brevior* (0.47%). Lungworm infections were strongly associated with younger age and showed marked seasonal variation, with higher prevalence in autumn and winter. Lung ultrasound consistently revealed diffuse B-line artifacts and other signs of reduced lung aeration, often in the absence of severe respiratory signs. Following treatment with topical imidacloprid/moxidectin, complete resolution of ultrasonographic abnormalities and clinical signs was observed. Conclusions: These findings confirm that feline pulmonary nematodes are present in Poland and may be underdiagnosed. Lung ultrasound represents a sensitive and non-invasive tool for detecting and monitoring lung involvement, but should be interpreted in conjunction with epidemiological data, parasitological results and therapeutic response.

## Simple Summary

Pulmonary nematodes are an important but often overlooked cause of respiratory disease in cats. These parasites may cause a wide range of clinical presentations, from mild or absent signs to severe breathing difficulties. Diagnosis is challenging because infected cats may not consistently shed parasite larvae in their feces, and routine diagnostic methods may fail to detect the infection. This study investigated the occurrence of feline lungworms in cats in Poland and evaluated lung ultrasound as a complementary diagnostic and monitoring tool. Fecal samples from over one thousand cats were examined to assess the prevalence of pulmonary nematodes. Additionally, a separate group of cats with suspected lung disease underwent lung ultrasound examinations both before and after treatment. Lungworms were commonly detected, particularly *Aelurostrongylus abstrusus*, and infections were more frequent in younger cats, during autumn and winter. Lung ultrasound often revealed extensive lung changes even in cats without obvious respiratory symptoms. Importantly, these ultrasound abnormalities completely resolved after treatment with a moxidectin-based anthelmintic. These findings show that lungworm infections are present in cats in Poland. Lung ultrasound is a sensitive but non-specific adjunct that may support clinical suspicion of pulmonary helminthiasis and is particularly useful for monitoring recovery after treatment; however, it should not be used as a stand-alone diagnostic test and must be interpreted together with coprological/molecular results and clinical context and relevant patient information (e.g., age, lifestyle, indoor/outdoor status).

## 1. Introduction

Pulmonary nematode infections represent a significant and underappreciated cause of respiratory disease in domestic cats worldwide. Among these, *Aelurostrongylus abstrusus* (Railliet, 1898), *Troglostrongylus brevior* (Gerichter, 1949) and *Eucoleus aerophilus* (Creplin, 1839) are considered the most clinically relevant lungworms affecting companion animals, particularly cats. These parasites colonize different compartments of the respiratory tract, including bronchi, bronchioles, alveolar ducts and alveoli, where they induce inflammatory and structural changes that may result in a broad spectrum of clinical manifestations, ranging from subclinical infection to severe, life-threatening respiratory compromise [1,2]. Metastrongylid lungworms, such as *A. abstrusus* and *T. brevior*, primarily inhabit bronchioles and alveolar ducts, where they induce interstitial and peribronchiolar inflammation and subpleural lesions. In contrast, adult *E. aerophilus* resides mainly within the tracheal and bronchial epithelium, causing chronic tracheobronchitis and epithelial damage. These differences may influence the distribution and character of imaging findings. When present, clinical signs are variable and non-specific, reflecting the extent of pulmonary involvement and the host’s immune response. Mild disease may manifest as occasional coughing, sneezing, or serous nasal and ocular discharge, whereas more pronounced respiratory involvement can lead to persistent cough, tachypnea, dyspnea and increased respiratory effort, including abdominal breathing. In severe cases, cats may develop marked respiratory distress, lethargy, exercise intolerance and potentially fatal respiratory failure. The variability and often subtle nature of clinical presentation frequently complicate early diagnosis and may contribute to under recognition of pulmonary nematode infections in feline patients [3,4,5]. *Aelurostrongylus abstrusus* is regarded as the most prevalent feline lungworm with reports from Europe. Epidemiological studies have demonstrated highly heterogeneous prevalence rates, ranging from sporadic cases to values exceeding 20–50% in endemic foci, depending on geographic region, climatic conditions and diagnostic methods applied [2,6]. According to published field surveys, the prevalence of *Troglostrongylus brevior* in cats varies geographically, but it is consistently reported in the Mediterranean Europe and surrounding regions. In Greece, *T. brevior* was identified in 7/125 (5.6%) stray cats [7]. In Jerusalem, lungworms were found in 40/400 (10.0%) feral cats, and 38/400 (9.5%) were shedding *T. brevior* [8]. In Romania, a retrospective multiplex PCR survey identified *T. brevior* DNA in 25/371 (6.7%) domestic cats [9]. Remarkably, an Italian study found that kittens 6 months of age or younger had a much higher prevalence of *T. brevior* infection [10]. Beyond metastrongyloid lungworms, cats may also develop respiratory disease due to trichuroid nematode *Eucoleus aerophilus*. *E. aerophilus* differs from metastrongyloid lungworms in both its life cycle and localization. Adult worms reside in the tracheal and bronchial epithelium, causing chronic inflammation and epithelial damage. Although historically considered sporadic, increasing evidence suggests that pulmonary eucoleosis is underdiagnosed and may be more prevalent than previously assumed, particularly in animals with outdoor access or immunocompromising conditions [11,12]. Most lungworm nematodes affecting cats rely on indirect life cycles that involve intermediate hosts such as snails and slugs. Infections caused by species such as *A. abstrusus* are associated with exposure to gastropods. In contrast, *E. aerophilus* may infect the definitive host either directly from the environment or through earthworms, which act as facultative intermediate hosts. Consequently, the risk of infection in companion animals is influenced not only by the presence of suitable vectors but also by their abundance and the local prevalence of parasitic infection [2].

Despite growing awareness, diagnosing pulmonary nematode infections remains challenging. Copromicroscopic examination using the Baermann technique remains the reference method for detecting first-stage larvae of metastrongyloid lungworms. However, its sensitivity is limited by intermittent larval shedding, low parasite burdens, prepatent infections, and the need for repeated sampling. According to the study, pharyngeal swabs represent the most reliable specimen for molecular diagnostic methods, especially PCR-based assays, provide improved sensitivity and specificity, and enable species differentiation. Comparative studies have demonstrated that PCR detects lungworm DNA in a substantially higher proportion of infected cats than copromicroscopy alone, including cases with negative fecal examinations [11,13]. Nevertheless, molecular diagnostics and invasive respiratory sampling, such as bronchoalveolar lavage (BAL), are not routinely available in many clinical settings [14]. In select clinical cases, diagnosis of *A. abstrusus* verminous pneumonia has been achieved through ultrasound-guided fine-needle aspiration of affected lung parenchyma in a cat [15].

Thoracic radiography and computed tomography are widely used imaging modalities for evaluating pleural and pulmonary disorders, with lung ultrasonography increasingly recognized as a valuable complementary diagnostic tool. Radiographic findings are frequent but nonspecific and include bronchial, interstitial, alveolar, interstitial-nodular pattern and mixed patterns, often with diffuse or multifocal distribution [3,4,16,17]. Importantly, radiographic severity does not consistently correlate with clinical severity or parasite burden, though most of the infections are asymptomatic [1,17]. Computed tomography (CT) also enables the characterization of pulmonary lesions, revealing nodules, peribronchial thickening, ground-glass opacities and lymphadenopathy that may not be visible on radiographs [5,18]. Lung ultrasound (LUS) has emerged as a valuable adjunctive imaging modality for assessing pulmonary disease in small animals. In companion animals with aelurostrongylosis, LUS frequently reveals increased and confluent B-lines, pleural line irregularities, subpleural nodules and focal lung consolidations. Although nonspecific, these findings indicate increased lung density and reduced aeration [14,19,20]. Histopathological studies provide a mechanistic explanation for these ultrasound artifacts. Pulmonary nematode infections induce interstitial inflammation, alveolar flooding with inflammatory exudate, eosinophilic and granulomatous reactions, and peribronchial infiltrates, all of which disrupt normal air–tissue interfaces and generate B-lines [18]. Parasite-induced changes are reversible, lung ultrasound offers a functional means of monitoring treatment response through the gradual resolution of B-line artifacts following effective anthelmintic therapy. Macrocyclic lactones and combination products, including imidacloprid/moxidectin (Advocate^®^, Bayer, Leverkusen, Germany) and eprinomectin-containing formulations (Broadline^®^, Merial, Lyon, France), have demonstrated high efficacy against feline lungworms. Field and experimental studies reported complete cessation of larval shedding and clinical improvement following treatment [21,22,23].

### Aim of the Study

This study comprised two independent cohorts. The first aim was to estimate the prevalence of feline pulmonary nematodes in Poland based on coprological examination, supported by molecular species confirmation. The second aim was to characterize lung ultrasound (LUS) abnormalities in a separate clinical cohort of cats with suspected pulmonary disease and to document the evolution of LUS findings following moxidectin-based anthelmintic treatment.

## 2. Materials and Methods

### 2.1. Study Design

This study was designed as a combined coprological and observational clinical investigation comprising two independent animal cohorts. The first cohort consisted of cats examined for pulmonary nematode infections using coprological and molecular diagnostic methods. The second cohort included a separate group of cats that underwent lung ultrasound examination as part of their clinical evaluation for suspected pulmonary disease. No animal was intentionally exposed or experimentally infested for the purposes of this study, and all procedures were performed as part of routine diagnostic or therapeutic management.

### 2.2. Population Study

A total of 1058 cats from Poland were examined. Age ranged from 1 month to 18 years, with a median age of 5 years (IQR 2.00–8.31); males accounted for 628/1058 (59.36%) and females for 430/1058 (40.64%). By age strata, the cohort comprised 89 (8.41%) cats aged < 6 months, 65 (6.14%) aged 6–11 months, 100 (9.45%) aged 12–23 months, 253 (23.91%) aged 2–4.9 years, and 551 (52.08%) aged ≥ 5 years (Table 1). Samples originated from 13 voivodeships, predominantly Masovian (36.48%) and Lower Silesia (16.07%).

### 2.3. Coprological Study Population and Examination

The coprological part of the study included fecal samples from 1058 client-owned cats presented to veterinary clinics in Poland collaborating with the commercial veterinary laboratory Animallab (Warsaw, Poland) between January and December 2025. Cats originated from multiple voivodeships. Age and sex data were available for all cats, whereas breed and lifestyle category (indoor/outdoor/stray) were not recorded. From each cat, feces were collected as a pooled sample from three consecutive days and submitted as a single diagnostic specimen.

Motile first-stage nematode larvae were isolated from fresh feces using the Baermann technique (5–10 g; incubation at room temperature for ≥12 h) and examined microscopically at 100× and 400× magnification [24]. Eggs of *E. aerophilus* were detected using high-specific-gravity zinc sulfate centrifugal flotation (750× *g* for 5 min; coverslip 25 min), and the same method was used for routine detection of helminth eggs and protozoan cysts/oocysts [25,26]. First-stage larvae were differentiated using standard morphological criteria and parasitological keys, with particular attention to distinguishing *Aelurostrongylus abstrusus* and *Troglostrongylus brevior*. Species-level identification based on morphology alone was considered presumptive and was confirmed by duplex PCR in available samples [2]. For *E. aerophilus*, diagnosis was based on the presence of characteristic barrel-shaped eggs with bipolar plugs [12].

### 2.4. Molecular Diagnostics

DNA from lungworm larvae identified in the Baermann sediment, was extracted using the TANBead Maelstrom 4800 Nucleic Acid Extraction System (Taiwan Advanced Nanotech Inc., Taoyuan City, Taiwan). The extracted DNA was stored at −20 °C until further analyses. A single-step duplex PCR targeting the ribosomal internal transcribed spacer 2 (ITS-2) region was used for simultaneous detection and differentiation of *A. abstrusus* and *T. brevior*, as previously described [27]. The assay employs two species-specific forward primers and a shared reverse primer: TrogloF (5′-GCACTTGAAATCTTCGACA-3′) for *T. brevior*, AeluroF (5′-GCATTTATGCTAGTGATATC-3′) for *A. abstrusus*, and MetR (5′-TAAGCATATCATTTAGCGG-3′). PCR was performed in a 50 µL reaction mixture containing 25 µL of StartWarm HS-PCR Mix (A&A Biotechnology, Gdańsk, Poland), 2 µL of each primer (5 µM), 3 µL of template DNA, and ultrapure water (A&A Biotechnology, Gdańsk, Poland) added to a final volume of 50 µL. Thermal cycling conditions comprising initial activation at 95 °C for 3 min, followed by 40 cycles of 95 °C for 1 min, 50 °C for 1 min, and 72 °C for 1 min, with a final extension at 72 °C for 7 min. Amplicons were resolved by agarose gel electrophoresis. Expected product sizes were 220 bp for *A. abstrusus* and 370 bp for *T. brevior*. Molecular diagnostics were performed on metastrongyloid-positive samples detected by Baermann examination (N = 88), to confirm species identity.

### 2.5. Lung Ultrasound Study Population

The lung ultrasound study population comprised seven client-owned cats that were either presented with suspected pulmonary disease or nonspecific clinical signs, or were diagnosed incidentally. This cohort was analyzed separately from the coprological study population to avoid methodological overlap and enable an independent assessment of imaging findings.

### 2.6. Lung Ultrasound Examination

Lung ultrasound examinations were performed using a portable Mindray M9 ultrasound (Mindray, Shenzhen, China) equipped with a L12-4s linear transducer. Animals were examined in a standing or sternal position without sedation. In all cases, follow-up lung ultrasound examinations were performed 2 months after the first treatment dose to assess the evolution of pulmonary abnormalities. Resolution or reduction of B-lines and other ultrasonographic changes were documented during clinical follow-up. A standardized scanning protocol was applied, including bilateral examination of cranial, middle and caudal lung fields in both dorsal and ventral thoracic regions. The pleural line was evaluated for continuity and regularity, and the presence, number and distribution of B-lines were recorded. B-lines were defined as vertical, hyperechoic reverberation artifacts arising from the pleural line, extending to the bottom of the ultrasound screen and moving synchronously with respiration. Additional ultrasonographic findings, such as subpleural nodules, lung consolidation, pleural effusion, and pleural line irregularities, were documented when present.

### 2.7. Echocardiography

All cats underwent transthoracic echocardiographic examination using a Mindray M9 ultrasound system (Mindray, Shenzhen, China) equipped with a phased-array cardiac transducer (7–11 MHz). Echocardiography was performed in all cats included in the lung ultrasound cohort (n = 7) to exclude cardiogenic causes of B-line artifacts. Examinations were performed without sedation, with cats gently restrained in a standing position. Standard two-dimensional, M-mode and Doppler echocardiographic views were obtained according to current guidelines. Particular attention was paid to the assessment of tricuspid and pulmonary valve regurgitation using continuous-wave Doppler, with peak regurgitant velocities measured when present. These measurements were used to estimate pulmonary arterial pressure and to screen for pulmonary hypertension. All measurements were obtained from optimized views.

### 2.8. Coprological Examination

Coprological examination in this cohort was performed using the same methodology as described for the coprological study population above.

### 2.9. Treatment and Follow-Up

Animals with suspected pulmonary nematode infection based on clinical assessment, imaging findings, epidemiological risk factors, or diagnostic results were treated with topical anthelmintic formulations containing moxidectin (imidacloprid/moxidectin, Advocate^®^, Bayer, Leverkusen, Germany) administered twice at a one-month interval.

### 2.10. Statistical Analysis

All statistical analyses were performed in R (R Foundation for Statistical Computing, Vienna, Austria; version 4.x) using two-tailed tests and an a priori significance threshold of *p* < 0.05. Continuous variables (age) were summarized using the median and interquartile range (IQR), whereas categorical variables were summarized as counts and percentages. Parasite prevalence was reported as proportions with 95% confidence intervals (CI) calculated using the Wilson method. Associations between parasite detection (binary outcomes: *T. brevior*, *A. abstrusus*, *E. aerophilus* and “any positive”) and explanatory variables were assessed using Pearson’s chi-square test or Fisher’s exact test where appropriate due to sparse counts for categorical predictors and the Mann–Whitney U test for age comparisons. For epidemiological stratification, age was additionally categorized into <6 months, 6–11 months, 12–23 months, 2–4.9 years and ≥5 years, and season was defined meteorologically as winter (Dec–Feb), spring (Mar–May), summer (Jun–Aug) and autumn (Sep–Nov). Multivariable associations were estimated using binary logistic regression models including age (per 1-year increase), sex (male vs. female) and season (reference: winter), and results were expressed as odds ratios (OR) with 95% CI.

## 3. Results

Duplex PCR was performed on n = 81 metastrongyloid-positive samples. The assay confirmed *A. abstrusus* in n = 76 (220 bp) and *T. brevior* in n = 5 (370 bp) samples. No discordant results were observed between microscopy-based identification and PCR. The distribution of examined feline fecal samples across Polish voivodeships, with the voivodeship-specific prevalence of detected lungworms, is listed in Table 2.

**Figure 1:**
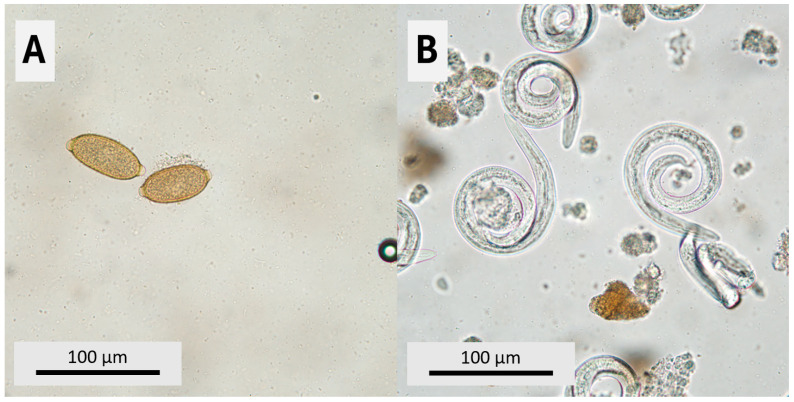
Respiratory nematodes stages detected in the present study: (**A**) eggs of *Eucoleus aerophilus* detected by high-specific-gravity zinc sulfate flotation method; (**B**) first-stage metastrongylid larvae (L1) isolated from feces using the Baermann technique.

Overall, 104/1058 cats were positive for ≥1 lungworms (9.83%, 95% CI 8.18–11.77); *A. abstrusus* was most frequent (7.18%, 95% CI 5.78–8.90), followed by *E. aerophilus* (2.17%, 95% CI 1.45–3.24) and *T. brevior* (0.47%, 95% CI 0.20–1.10) (Figure 1, Table 3). No co-infections were observed (all positives had exactly one parasite detected). Positivity was strongly age-dependent (χ^2^ tests by age group: “any positive” χ^2^ = 225.53, df = 4, *p* < 0.001; *A. abstrusus* χ^2^ = 248.29, df = 4, *p* < 0.001; *E. aerophilus* χ^2^ = 13.86, df = 4, *p* = 0.0078), peaking in kittens aged 6–11 months (any positive 52.31%; *A. abstrusus* 49.23%); *T. brevior* was detected exclusively in cats <6 months (Table 4). Seasonality was evident for “any positive” and *A. abstrusus* (any χ^2^ = 48.73, df = 3, *p* < 0.001; *A. abstrusus* χ^2^ = 44.53, df = 3, *p* < 0.001), with the highest prevalence in winter (Table 5); month-level differences were also significant (any χ^2^ = 67.18, df = 11, *p* < 0.001; *A. abstrusus* χ^2^ = 77.43, df = 11, *p* < 0.001). In multivariable logistic regression, increasing age was independently associated with lower odds of detection for “any positive” (OR per +1 year = 0.591, *p* < 0.001), *A. abstrusus* (OR = 0.519, *p* < 0.001) and *E. aerophilus* (OR = 0.822, *p* = 0.008). After adjustment, males had lower odds of “any positive” (OR = 0.533, *p* = 0.007) and *E. aerophilus* (OR = 0.423, *p* = 0.048), and spring/summer/autumn were associated with reduced odds versus winter for “any positive” and *A. abstrusus* (Table 6).

### 3.1. Lung Ultrasound Study Population Results

The lung ultrasound study population included seven client-owned cats aged between 6 months and 1.5 years, comprising four females and three males. Five cats had been adopted from animal shelters and were considered to have a potential exposure to lungworms during that period, while two cats originated from household environments but had outdoor access.

Four cats presented with mild clinical signs, including sneezing and serous nasal discharge. Two cats showed no overt clinical abnormalities; however, based on previous experience, their owners elected to pursue screening echocardiographic and lung ultrasound examinations. One cat was examined incidentally during a pre-anesthetic echocardiographic evaluation, during which numerous B-lines were observed in the pericardiac region. Consequently, the owner was advised to extend the cardiac examination to include a complete lung ultrasound assessment.

### 3.2. Lung Ultrasound Examination Results

In all 7 cats, lung ultrasound revealed a marked interstitial pattern characterized by numerous B-lines. B-lines were observed in approximately 40–70% of thoracic scanning sites, indicating diffuse pulmonary parenchymal involvement. The distribution of B-lines was bilateral and not confined to a single lung region.

In two cats, additional abnormalities of the pleural line were identified, consisting of pleural line thickening with reduced regularity. In one cat, small subpleural consolidations were detected, measuring up to 1 mm in thickness. No large areas of consolidation, pleural effusion, or focal alveolar patterns were observed in any of the examined cats.

At follow-up examination after treatment, all cats demonstrated a lung ultrasound pattern consistent with normal, well-aerated lungs across all examined scanning windows. In every intercostal region assessed, the pleural line appeared thin, smooth and regular, with preserved pleural sliding. Only physiological reverberation artifacts were observed, including horizontal A-lines, whereas pathological artifacts, such as B-lines or subpleural consolidations, were absent. No focal or diffuse ultrasonographic abnormalities were detected in any lung region.

### 3.3. Echocardiography Results

Echocardiographic examination revealed no evidence of structural or functional cardiac abnormalities in any of the 7 cats. No valvular insufficiencies were detected, and there were no signs of cardiac chamber enlargement. Left and right ventricular dimensions, as well as systolic function, were within physiological reference ranges in all examined animals.

### 3.4. Coprological Examination Results

Coprological examination revealed the presence of *A. abstrusus* larvae in four of the seven examined cats. Identification was based on the characteristic morphological features of first-stage larvae (L1) observed in fecal samples. The remaining three cats tested negative for lungworm larvae in coprological analysis.

## 4. Discussion

### 4.1. Epidemiology and Prevalence of Feline Pulmonary Nematode Infections in Poland

The present study provides complementary epidemiological and clinical data on pulmonary nematode infections in cats in Poland, with particular focus on diagnostic constraints, lung ultrasonographic features and responses to anthelmintic treatment. The results confirm that *A. abstrusus*, *T. brevior* and *E. aerophilus* are present in companion animals in Poland, supporting the growing body of evidence that pulmonary nematodes are endemic across large parts of Europe [2,28]. These findings are consistent with broader European data. A large multicenter survey across 12 European countries reported an overall lungworm prevalence of 20.8% (range: 0.8–35.8%), with *A. abstrusus* as the predominant species [29]. The 7.18% prevalence of *A. abstrusus* in our study is higher than the low single-digit levels frequently reported from routine diagnostic submissions in parts of Europe, yet it remains within the wide range described in continental datasets and reviews [29,30,31]. Copromicroscopic detection is dependent on the sampling strategy and larval output, so cross-study comparisons should be conducted with caution. Exclusive testing of symptomatic or higher-risk cats may inflate apparent prevalence, while low larval burdens, intermittent shedding, and poor Baermann execution can produce false-negative results [29,31].

### 4.2. Methodological Considerations and Cross-Study Comparability

The prevalence reported in the present study is higher than that documented in previous investigations conducted in southern Poland. However, this discrepancy is likely attributable to substantial methodological differences, as those studies were based primarily on necropsy findings and cytological examination of respiratory samples. The use of different diagnostic materials and approaches, each with distinct sensitivities and inherent limitations, may therefore partly explain the observed variation in reported prevalence and underscores the challenge of directly comparing epidemiological data derived from heterogeneous study designs [28]. Furthermore, the prevalence of *A. abstrusus* identified in the present study exceeds that reported in a copromicroscopic survey conducted in south-eastern Poland, in which lungworm larvae were detected in 1.1% of examined cats (January 2016–April 2019) [32]. Although both studies relied on fecal examination techniques, including the Baermann method, several factors may account for the higher prevalence observed in our dataset. Differences in the geographical coverage, study period, population structure and risk profile of the sampled cats, as well as variations in sampling intensity and diagnostic workflows, may have influenced detection rates. In particular, age distribution is likely to be relevant, as lungworm infections have consistently been shown to occur more frequently in younger animals. These findings further support the notion that the epidemiology of feline lungworm infections may vary substantially between regions and populations, even when similar diagnostic approaches are applied, and underscore the importance of continued regional surveillance. In conjunction with our nationwide findings, these sources indicate that *A. abstrusus* is likely underdiagnosed when Baermann examination is not conducted routinely and when coprology is limited to flotation-only screening [28,31]. These findings lend credence to the idea that feline lungworm are not limited to traditional “endemic Mediterranean” settings but rather occur more widely throughout Europe [7,29,31,33].

### 4.3. Age-Related Patterns and Potential Transmission Routes

The high prevalence in our study may be attributed to the large sample size and coverage of a wide area, the inclusion of cats that visited the vet, and the methodological sensitivity of examining pooled feces, which is often recommended to minimize day-to-day fluctuations in larval shedding [31].

Despite the low overall prevalence of *T. brevior* in the present study (0.47%), a clear epidemiological pattern was observed, with infections occurring exclusively in cats younger than 6 months of age. The established clinical-epidemiological profile of *T. brevior* as a lungworm of particular concern in pediatric feline patients, in whom infection may be severe and occasionally fatal, is strongly supported by this [34]. It is hard to explain the age limit seen here with “normal” indirect transmission alone, since kittens 2–4 months old often do not have much independent predatory exposure. Instead, our findings are compatible with early-life acquisition, including mother-to-offspring routes [35].

A higher prevalence in younger cats is consistent with increased exposure early in life (including predation on intermediate or paratenic hosts), a lack of acquired immunity, and a higher probability of prepatent or low-burden infections with intermittent shedding. Importantly, the traditional view that *T. brevior* infection is acquired exclusively through ingestion of intermediate or paratenic hosts has been challenged by reports of vertical/perinatal transmission, including transmission from queens to nursing kittens [36,37]. Although transplacental transmission is difficult to document and remains debated, the consistently higher occurrence of *T. brevior* in very young kittens across multiple studies supports the notion that vertical/perinatal routes may play a relevant role in some cases [34,36,37]. The findings of the present study support the practical recommendation that veterinarians should maintain a high index of suspicion for *T. brevior* infection in young cats presenting with respiratory disease, even outside traditionally recognized endemic areas. This is substantiated by the exclusive occurrence of *T. brevior* infections in kittens and the observed winter clustering in the seasonal distribution pattern [34,36,37].

### 4.4. Species-Specific Findings: Eucoleus aerophilus and Seasonality

*E. aerophilus* eggs were detected in 2.17% of cats. This prevalence was similar to the pooled prevalence reported in a systematic review and meta-analysis of cat fecal samples and higher than that in lung necropsy [38]. Epidemiologically and diagnostically, the distinction between lung and fecal prevalence is significant. Due to the intermittent shedding of eggs in feces, fecal examination may underestimate the burden of *E. aerophilus* infection. Additionally, coprological examination has inherent limitations when the number of eggs is low. The diagnosis of *E. aerophilus* in stray cats in Poland confirms that this parasite circulates in the feline population, and domestic cats can be exposed to it in environments shared with wildlife reservoirs, such as foxes [39]. Consequently, the observed prevalence is biologically plausible and consistent with both global syntheses and observations from Poland [38,39].

Seasonality emerged as a prominent epidemiological feature of *A. abstrusus* infections in the present study, with the highest proportion of positive cases recorded during winter and an increasing trend observed in autumn. In contrast, detections of *T. brevior* were predominantly confined to the winter period, whereas *E. aerophilus* showed less pronounced seasonal variation. The seasonal character of parasite detection may be correlated with several non-mutually exclusive mechanisms, including the ecology of intermediate hosts and paratenic hosts, temporal dynamics of infection and patency, and diagnostic sampling bias. Because *A. abstrusus* has an indirect life cycle and larval excretion can be low or intermittent, winter peaks in fecal detection can plausibly arise from infections acquired during periods of higher intermediate-host activity, with patent shedding and clinical presentation falling into subsequent months [31,40]. Moreover, methodological factors, including day-to-day variability in larval shedding, can modulate observed seasonality, again highlighting the rationale for pooled multi-day fecal sampling when feasible [31]. In general, our seasonal patterns indicate that surveillance limited to a single time window may yield inaccurate estimates of disease prevalence. We recommend testing throughout the year, including the Baermann examination, when necessary, especially in kittens and adult outdoor cats [31,34,40].

### 4.5. Diagnostic Integration and Clinical Interpretation: The Role of Lung Ultrasound

Coprological examination in the population cohort did not reveal co-detection of *A. abstrusus* with *T. brevior* or *E. aerophilus*, despite the occurrence of mixed cardiopulmonary and intestinal parasitic infections in European cats [7,29,34]. This may be attributed to the low prevalence of *T. brevior* or possible true ecological separation. Nevertheless, it is more likely that the failure of coproscopy to identify concurrent low-level infections is the main cause. The initial stages of feline lungworms are morphologically similar, so care must be taken when identifying species through microscopy. When clinical and epidemiological decisions rely on precise species identification, molecular methods should be employed [29,31,34]. Importantly, the coprological data presented worldwide suggest that a proportion of lungworm infections may remain undetected due to biological and methodological constraints, despite ongoing pulmonary pathology. This observation is particularly relevant in the context of subclinical infections, where respiratory signs may be absent or mild. Consequently, imaging-based techniques that visualize lung alterations directly, such as lung ultrasound, warrant evaluation as adjunctive diagnostic tools, as explored in the subsequent analysis of a distinct clinical cohort. One of the most striking observations of the present study was the frequent lack of correlation between the severity of lung ultrasound abnormalities and the clinical respiratory status of affected animals. In the lung ultrasound cohort, extensive and diffuse B-lines were detected in multiple thoracic regions in all animals, often involving numerous scanning windows. Despite these advanced ultrasonographic findings, the majority of animals did not exhibit dyspnea. Similar observations have previously been reported using thoracic radiography. In a radiographic study of cats infected with pulmonary nematodes, no significant correlation was found between clinical severity scores and radiographic findings, and several subclinically lungworm infection displayed marked parenchymal lung alterations despite the absence of overt respiratory signs [16]. These findings collectively suggest that both ultrasonographic and radiographic abnormalities may precede or exceed the clinical expression of disease, highlighting the limited predictive value of imaging severity alone for assessing clinical respiratory compromise in feline lungworm infections. Lungworm infections predominantly induce interstitial and peribronchiolar inflammation, often with subpleural localization and granulomatous reactions around eggs and larvae [18]. These changes increase lung density locally and disrupt air-tissue interfaces, leading to the generation of B-lines on lung ultrasound. Only 4 animals in the lung ultrasound cohort had coprological confirmation of pulmonary nematode infection. This finding should not be interpreted as evidence against a parasitic etiology but rather reflects the well-documented diagnostic limitations of fecal examinations. Intermittent larval shedding, low parasite burdens and prepatent infections are common, particularly in young animals, and frequently result in false-negative Baermann results [13]. Notably, all animals included in the study showed complete resolution of severe ultrasonographic lung abnormalities within two months following administration of the first dose of topical imidacloprid/moxidectin (Advocate^®^, Bayer, Leverkusen, Germany), without the use of any additional pharmacological treatment. Follow-up lung ultrasound examinations revealed restoration of normal aerated lung patterns, characterized by a thin, regular pleural line and the absence of B-lines. This consistent and uniform response to targeted anthelmintic therapy provides strong clinical support for a parasitic origin of the observed pulmonary changes, even in cases with negative coprological findings. This observation aligns with previous reports describing favorable clinical and imaging outcomes following moxidectin treatment in cats with pulmonary nematode infections. Crisi et al. [3] reported rapid clinical improvement, with resolution of respiratory signs occurring as early as two weeks after the first dose in most cats. Normalization of thoracic radiographic findings was documented over a broader time frame, ranging from 2 to 6 weeks after treatment initiation [3]. In the present study, the timeframe to complete ultrasonographic normalization should be interpreted considering the follow-up protocol rather than as an indication of delayed recovery. Lung ultrasound examinations were performed at predefined intervals, and full resolution of abnormalities was documented at the scheduled follow-up, which occurred within two months after treatment initiation. Consequently, earlier normalization of lung ultrasound findings cannot be excluded, but was not assessed due to the study design. Collectively, these observations support the use of lung ultrasound as a valuable tool for monitoring pulmonary recovery following anthelmintic treatment, while underscoring the importance of interpreting imaging findings in relation to the timing of post-treatment evaluations. Collectively, these observations emphasize that a negative fecal examination does not exclude pulmonary nematode infection and that therapeutic response may represent an important adjunctive indicator of parasitic disease in clinical practice [41,42,43]. The generation and appearance of B-line artifacts in lung ultrasound are influenced by a wider range of factors than previously recognized. The presence of multiple B-lines reflects an increase in lung tissue density resulting from reduced aeration of the subpleural lung regions. This ultrasonographic finding is non-specific and may be associated with various pathological conditions, including cardiogenic pulmonary edema, focal or diffuse interstitial lung diseases, infectious processes and acute respiratory distress syndrome (ARDS). In this context, B-line artifacts may also be observed in parasitic lung diseases, where inflammatory infiltrates, interstitial involvement, or localized pulmonary lesions contribute to increased lung density and altered acoustic properties. Therefore, lung ultrasound findings must always be interpreted within a broad diagnostic framework, particularly in regions where parasitic infections of the respiratory system are prevalent [20,43]. Overall, previous studies have shown that radiographic findings associated with lungworms lack specificity and may overlap with imaging features observed in a wide range of pulmonary conditions, including fibrotic lung disease, pulmonary edema, fungal infections, asthma and pulmonary neoplasia [5]. This findings of the present study support the concept that lung ultrasound is a sensitive but non-specific tool that can reveal early or subclinical lung pathology, necessitating careful integration with epidemiological data, age, lifestyle and response to treatment. The normalization of imaging findings following anthelmintic treatment is consistent with previous studies documenting radiographic improvement after successful elimination of lungworms [3]. In this context, the absence of echocardiographic evidence of pulmonary hypertension in cats undergoing combined cardiac and lung ultrasound examination further supports a primary pulmonary rather than cardiogenic origin of the observed imaging abnormalities, in agreement with earlier studies [5,17]. Collectively, these results emphasize the importance of a multimodal diagnostic approach in feline lungworm infections, in which coprological, imaging and therapeutic response data are interpreted together to achieve accurate clinical assessment.

### 4.6. Clinical Implications and Limitations

The findings of the present study have several important clinical implications. First, pulmonary nematode infections should be considered in the differential diagnosis of young cats presenting with LUS abnormalities in Poland, even in the absence of respiratory distress or coprological confirmation. Second, LUS may serve as a valuable adjunctive tool for monitoring treatment response, particularly in cases where fecal diagnostics are negative or inconclusive. Importantly, LUS findings, including B-lines, are non-specific and may overlap with a range of pulmonary conditions. Therefore, LUS should be regarded as an adjunct for detecting lung involvement and for monitoring response to therapy, rather than as a primary diagnostic method. In cats with suggestive LUS patterns and relevant risk factors, repeated coprology and/or molecular testing should be considered. This study has several limitations. First, the coprological and lung ultrasound cohorts were analyzed separately, which precluded direct correlation between parasitological status and imaging findings within the same large group of animals. Second, the timing of follow-up lung ultrasound examinations was predefined and aimed primarily at confirming complete resolution of abnormalities rather than characterizing the precise temporal dynamics of ultrasonographic recovery; therefore, earlier normalization of lung ultrasound findings may have occurred but was not assessed. Third, detailed data on the cats’ lifestyle and previous living conditions were unavailable; consequently, prior exposure to pulmonary nematode infection before enrollment cannot be excluded. Finally, we agree that copromicroscopy remains the first-line diagnostic approach. Our intention was not to downplay coproscopy, but to highlight its known sensitivity constraints in low-burden, intermittent, or prepatent infections, and to propose lung ultrasound as an adjunct for assessing lung involvement and for monitoring response to therapy.

## 5. Conclusions

In conclusion, pulmonary nematode infections caused by *A. abstrusus*, *T. brevior* and *E. aerophilus* are present in cats in Poland and are likely underdiagnosed due to limitations of fecal-based diagnostic methods. Lung ultrasound frequently reveals extensive pulmonary involvement characterized by numerous B-lines, even in clinically stable animals without dyspnea. The complete resolution of imaging abnormalities following treatment with imidacloprid/moxidectin alone strongly supports a parasitic etiology of these changes.

## Figures and Tables

**Table 1:** Study population characteristics (N = 1058).

Characteristic	Value
Total cats examined, N	1058
Sex, female, N (%)	430 (40.64%)
Sex, male, N (%)	628 (59.36%)
Age (years), range	0.08–18.00
Age (years), median (IQR)	5.00 (2.00–8.31)
Age group < 6 months, N (%)	89 (8.41%)
Age group 6–11 months, N (%)	65 (6.14%)
Age group 12–23 months, N (%)	100 (9.45%)
Age group 2–4.9 years, N (%)	253 (23.91%)
Age group ≥ 5 years, N (%)	551 (52.08%)

**Table 2:** Geographic origin of examined cats by voivodeships, with parasite detection by voivodeship.

Voivodeship	N	% of All Samples	Any (≥1) n (%)	*A. abstrusus* n (%)	*E. aerophilus* n (%)	*T. brevior* n (%)
Masovian	386	36.5	49 (12.7)	34 (8.8)	10 (2.6)	5 (1.3)
Lower Silesian	170	16.1	16 (9.4)	11 (6.5)	5 (2.9)	0 (0.0)
Greater Poland	108	10.2	8 (7.4)	6 (5.6)	2 (1.9)	0 (0.0)
Lesser Poland	93	8.8	9 (9.7)	9 (9.7)	0 (0.0)	0 (0.0)
Silesian	73	6.9	7 (9.6)	4 (5.5)	3 (4.1)	0 (0.0)
Podlaskie	51	4.8	5 (9.8)	4 (7.8)	1 (2.0)	0 (0.0)
Subcarpathian	38	3.6	1 (2.6)	1 (2.6)	0 (0.0)	0 (0.0)
Kuyavian–Pomeranian	32	3.0	1 (3.1)	1 (3.1)	0 (0.0)	0 (0.0)
Pomeranian	29	2.7	2 (6.9)	1 (3.4)	1 (3.4)	0 (0.0)
West Pomeranian	23	2.2	0 (0.0)	0 (0.0)	0 (0.0)	0 (0.0)
Lubusz	22	2.1	0 (0.0)	0 (0.0)	0 (0.0)	0 (0.0)
Warmian–Masurian	20	1.9	3 (15.0)	2 (10.0)	1 (5.0)	0 (0.0)
Lublin	13	1.2	3 (23.1)	3 (23.1)	0 (0.0)	0 (0.0)
Total	1058	100.0	104 (9.8)	76 (7.2)	23 (2.2)	5 (0.5)

**Table 3:** Overall parasite prevalence with Wilson 95% confidence intervals.

Outcome	Positive	Prevalence (95% CI)
*Troglostrongylus brevior*	5	0.47% (0.20–1.10)
*Aelurostrongylus abstrusus*	76	7.18% (5.78–8.90)
*Eucoleus aerophilus*	23	2.17% (1.45–3.24)
Any (≥1 detected)	104	9.83% (8.18–11.77)

**Table 4:** Prevalence of detected lungworms by age group.

Age Group	Any (≥1)	*A. abstrusus*	*E. aerophilus*	*T. brevior*
<6 months	28/89 (31.46%)	21/89 (23.60%)	2/89 (2.25%)	5/89 (5.62%)
6–11 months	34/65 (52.31%)	32/65 (49.23%)	2/65 (3.08%)	0/65 (0.00%)
12–23 months	12/100 (12.00%)	9/100 (9.00%)	3/100 (3.00%)	0/100 (0.00%)
2–4.9 years	23/253 (9.09%)	11/253 (4.35%)	12/253 (4.74%)	0/253 (0.00%)
≥5 years	7/551 (1.27%)	3/551 (0.54%)	4/551 (0.73%)	0/551 (0.00%)

**Table 5:** Prevalence of detected lungworms by season.

Season	Any (≥1)	*A. abstrusus*	*E. aerophilus*	*T. brevior*
Winter	59/336 (17.56%)	47/336 (13.99%)	8/336 (2.38%)	4/336 (1.19%)
Spring	9/218 (4.13%)	5/218 (2.29%)	4/218 (1.83%)	0/218 (0.00%)
Summer	2/199 (1.01%)	1/199 (0.50%)	1/199 (0.50%)	0/199 (0.00%)
Autumn	34/305 (11.15%)	23/305 (7.54%)	10/305 (3.28%)	1/305 (0.33%)

Abbreviation: seasons were defined as winter (Dec–Feb), spring (Mar–May), summer (Jun–Aug), autumn (Sep–Nov). Global association by chi-square was significant for Any (*p* < 0.001) and *A. abstrusus* (*p* < 0.001) but not for *E. aerophilus* (*p* = 0.208).

**Table 6:** Multivariable logistic regression (OR with 95% CI) for parasite detection.

Predictor	Any (≥1) OR (95% CI)	Any (≥1) *p*	*A. abstrusus* OR (95% CI)	*A. abstrusus p*	*E. aerophilus* OR (95% CI)	*E. aerophilus p*
Age (per 1-year increase)	0.591 (0.520–0.670)	3.543 × 10^−16^	0.519 (0.432–0.623)	2.352 × 10^−12^	0.822 (0.711–0.950)	0.008051
Male (vs. female)	0.533 (0.336–0.844)	0.007309	0.704 (0.415–1.193)	0.192	0.423 (0.180–0.992)	0.04785
Spring (vs. winter)	0.251 (0.117–0.540)	0.00041	0.190 (0.071–0.508)	0.0009228	0.929 (0.272–3.170)	0.9065
Summer (vs. winter)	0.063 (0.015–0.270)	0.0001903	0.044 (0.006–0.329)	0.002356	0.288 (0.035–2.353)	0.2457
Autumn (vs. winter)	0.398 (0.240–0.660)	0.0003578	0.326 (0.184–0.580)	0.0001362	1.268 (0.489–3.291)	0.6255

## Data Availability

The data presented in this study are available on reasonable request from the corresponding author. The data are not publicly available due to privacy restrictions.

## Abbreviations

The following abbreviations are used in this manuscript:

ARDS	acute respiratory distress syndrome
BAL	bronchoalveolar lavage
bp	base pairs
CI	confidence intervals
CT	computed tomography
DNA	deoxyribonucleic acid
IQR	interquartile range
ITS-2	internal transcribed spacer 2
LUS	lung ultrasound
OR	odds ratio
PCR	polymerase chain reaction
